# A pragmatic pilot randomized trial to investigate the effectiveness of behavioural activation group therapy in reducing depressive symptoms and improving quality of life in patients with depression: the BRAVE pilot trial protocol

**DOI:** 10.1186/s40814-015-0034-y

**Published:** 2015-11-10

**Authors:** Zainab Samaan, Kathryn Litke, Kathleen McCabe, Brittany Dennis, Jeff Whattam, Laura Garrick, Laura O’Neill, Terri Ann Tabak, Scott Simons, Sandra Chalmers, Brenda Key, Meredith Vanstone, Feng Xie, Gordon Guyatt, Lehana Thabane

**Affiliations:** 1 Department of Psychiatry and Behavioural Neuroscience, McMaster University, Hamilton, ON Canada; 2 Mood Disorders Research Unit, St. Joseph’s Healthcare Hamilton, Hamilton, ON Canada; 3 Department of Clinical Epidemiology & Biostatistics, McMaster University, Hamilton, ON Canada; 4 Population Genomics Program, Chanchlani Research Centre, McMaster University, Hamilton, ON Canada; 5 Department of Medicine, McMaster University, Hamilton, ON Canada; 6 Centre for Evaluation of Medicine, Hamilton, ON Canada; 7 System-Linked Research Unit, Hamilton, ON Canada; 8 Department of Anaesthesia, McMaster University, Hamilton, ON Canada; 9 Department of Paediatrics, McMaster University, Hamilton, ON Canada

**Keywords:** Pragmatic, Pilot, Protocol, Trial, Randomized, Behavioural, Activation, Group, Depression, Adult

## Abstract

**Background:**

Depression is a common disorder with a lifetime prevalence of 16 %. Despite the availability of several treatment options for depression, many patients do not respond to treatment and develop chronic illness associated with several secondary comorbidities. Behavioural activation (BA) is a simple therapy that has the potential for improving symptoms of depression and quality of life in patients with depression. The effectiveness of BA has not, however, been tested in a group format for patients with moderate to severe depression attending a specialized mood disorders tertiary care setting. Group format has the advantage of treating more patients at the same time especially in resource-limited settings. The primary objective of this pilot study is to test the feasibility of a main trial by assessing the recruitment and retention rates, average group size, completion of data and resources needed and receive the participants’ feedback on the intervention. The secondary objective is to explore the change in mood and quality of life measures in adults with depression receiving BA.

**Methods/Design:**

Using a pragmatic pilot randomized controlled trial design, we will test the feasibility of a large trial to assess the effectiveness of BA added to usual care compared to a depression support group with usual care. Participants will be randomized after obtaining informed written consent to one of two study arms. Face-to-face group therapy will be provided in a hospital setting by trained therapists. Intervention and control groups will be seen twice weekly for 10 weeks and then once weekly for further 8 weeks. Participants will be completing mood symptom scales, quality of life questionnaires and anthropometric measures and provide blood samples for future analysis of biomarkers of response to treatment. During the pilot study we will also solicit participants’ feedback and experience regarding the number, frequency and contents of the sessions as well as to explore participant perceptions of barriers or benefits associated with the BA program.

**Discussion:**

The pilot study will help to inform a larger trial and assist in modifying the intervention based on patients’ feedback.

**Trial registration:**

Clinicaltrials.gov Identifier NCT02045771.

Hamilton Integrated Research Ethics Board (HiREB) number: 14–042.

## Background

Depression is a common disabling disorder affecting 11.2 to 16.0 % of the general population [1, 2], and it is associated with a high risk of permanent disability (HR 1.48, 95 % CI 1.30, 1.69) and mortality (HR 2.50, 95 % CI 1.80, 3.48) [3].

Depression is a complex and potentially chronic disorder that requires several treatment interventions to achieve remission. Antidepressant medications have shown to be efficacious for some patients, but many patients develop troublesome side effects, and 42.7 % of patients show inadequate response to treatment [4, 5]. Studies have also shown that a large proportion of patients with depression (55.3 %) continue to have ongoing depressive symptoms at follow-up [6]. In addition, many patients prefer non-pharmacological interventions or report troublesome side effects. Therefore, investigating other treatment modalities is of critical value to the management of depression. Several psychotherapies have been shown to be effective in the management of depression alone or in combination with pharmacotherapy [7]. Cognitive behavioural therapy (CBT) [8] and behavioural activation (BA) [9] are considered as effective treatments for depression [10–12]; however, the evidence for BA therapy especially in a group format is less well-investigated compared to CBT. Although CBT is a commonly used treatment option for patients with depression, it may not be suitable for many patients as it requires a level of psychological insight in order to delve into complex understandings of core beliefs and behaviours. Additionally, an acceptance of psychological therapy and the motivation to change are also necessary for CBT treatment [13].

Group therapy has the advantage of delivering treatment to a larger number of patients within the same time that it takes to provide individual therapy with comparable benefits for many behavioural and cognitive forms of therapy [14]. Therefore, group therapy can be cost effective, reducing wait time and reaching a larger number of patients. In addition to the limited research on BA group therapy and depression, there is also a lack of research on the effectiveness of BA in improving quality of life, commonly affected in depression, in individuals with major depressive disorders [15].

The proposed behavioural activation program, *Out of the Blues*, will be tested for feasibility using a pilot study through a mixed method study including a pilot pragmatic randomized trial and a qualitative study component. The pilot trial will inform the design of a main trial. Pilot trials provide investigators the opportunity to assess feasibility, acceptability and cost of interventions intended to be tested on a large scale [16]. Pilot trials are not intended for testing intervention effectiveness but instead for evaluating important trial processes such as recruitment rate, attrition rate, potential for collaboration among international settings, as well as the acceptability of the intervention itself [16]. Important aspects of the intervention such as the use of interview rooms and practicality of using mobile computers to upload activity-tracking data require assessment prior to use in the full trial. Providing investigators an opportunity for process evaluation prior to trial initiation allows for the modification of critical flaws to the study design before these problems impact major study findings [16].

### Behavioural activation program summary: “Out of the Blues”

The *Out of the Blues* program’s primary goal is to reduce depressive symptoms and reintegrate patients more fully into their personal and professional lives thus improving quality of life and facilitating the attainment and maintenance of remission from depression. The Out of the Blues program includes several components to support individuals with depression reintegration to former activities as well as the acquisition of new skills in order to reduce the symptoms of depression, improve quality of life and achieve prolonged remission. The program components are centred on behavioural activation (BA) [9] with complementary interventions including recreation activities and behavioural modifications based on the principle that *what you do affects how you feel*. The complementary components aim to improve physical activity and promote a healthy lifestyle. They include skill building related to coping with stress and problem solving intended to improve overall health and wellbeing [17]. The Out of the Blues program begins with the behavioural monitoring of daily activities (using the daily activity record) by examining the participant’s activity and engagement levels with different aspects such as home, work, leisure and social activities. This will be followed by group therapy focused on encouraging participants to engage in activities that are identified by the participants through the activity record as personally important to them. As participants progress in the program, they will continue to monitor their activities, depressive symptoms and quality of life. Improvement in mood and quality of life at the end of the program will be measured using standardized instruments that are described in detail later in the protocol.

The Out of the Blues program will use a structured approach, including weekly face-to-face sessions, homework that includes recording activities (individualized activities that align with the participants’s values), rebuilding of individual skills or learning new skills in order to improve depressive symptoms and the quality of life of participants. If shown to be effective, the Out of the Blues program will be made available to all patients with depression attending the Mood Disorders Program, a tertiary care hospital-based centre. A multidisciplinary team of clinicians that includes psychiatrists, occupational therapists, recreational therapists and clinical psychologists designed this program. This program is intended to be administered by clinicians with training in any of these designations; however, the inclusion of an integrated team is preferable. Ultimately the goals of the program are to:

1. Reduce depressive symptoms to achieve remission of depressive disorders
2. Enhance patients’ strengths and skills to fulfil their goals in life as demonstrated by return to/or starting new work (at home activities or work place), education or volunteering for activities
3. Improve physical health by reducing unhealthy behaviours (example sedentary lifestyle)
4. Encourage social network building and community activities
5. Enhance the likelihood of prolonged remission by achieving the above goals

However, before embarking on the full program, a pilot study to assess the feasibility of delivering this new program for patients with depression at a tertiary hospital-based centre is needed. In this study protocol, we present the details of the planned pilot study.

### The choice of comparators for the pilot trial

#### The intervention (Out of the Blues), why behavioural activation?

##### Definition of behavioural activation

Originally a component of cognitive therapy, behavioural activation is the use of strategies such as activity scheduling, mastery/pleasure ratings and graded task assignments to change one’s perception of specific situations [9]. It involves the use of activities to improve life situations and depressed mood [9].

BA individual therapy has been shown to be effective in the treatment of depression compared to cognitive therapy, antidepressant and a placebo [9, 18–20]. BA was also found to be a flexible treatment that can be individualized to each patient [9]. A meta-analysis of 16 studies which included 780 participants (241 participants were in the BA intervention) [21] found a significant positive effect of activity scheduling, a component of behavioural activation, on reducing depressive symptoms with a relatively large effect size (0.87, 95 % CI 0.6, 1.15). This effect size is comparable to treatment effects seen in other treatment modalities for depression including antidepressant medications [22, 23]. This meta-analysis combined studies of BA provided in group (*n* = 4) or individual format (*n* = 12) of varying numbers of session from as low as 4 to as high as 20 sessions [21] and included only one component of BA (activity scheduling).

The number of studies using BA in a group format is limited to a small number of participants and short follow-up duration. In addition, the majority of patients included in these studies suffered from mild to moderate depressive symptoms in community-based treatment settings. We will test the effectiveness of BA in a representative sample of patients with depression attending a tertiary care hospital setting using a pragmatic study design. The BA intervention will be delivered in a group of 6–12 participants as an adjunct therapy to usual care. Usual care is currently received at the Mood Disorders Program and includes pharmacotherapy, psychotherapy, occupational therapy, case management and regular follow-up visits to the clinic. No structured BA intervention is currently offered at this tertiary mood disorders clinic. While we acknowledge that studies often provide “standard of care,” as the comparator arm, we are reluctant to use this term when defining our own comparator. Contention exists in the medical community over use of the term “standard of care”, which stems largely from the lack of consensus as to (1) what constitutes appropriate use of the term, (2) what expertise is required to declare an intervention to the standard of care, and (3) what level of evidence is adequate to support or refute a therapy as the standard [24]. In fact, researchers suggest that the term not be adopted for any intervention unless confirmatory randomized controlled trials or meta-analyses exist to support this declaration [24]. Recognizing that there is limited evidence to suggest that what is provided to patients in our comparator arm fulfils the criteria for “standard of care,” we have elected to use the term “usual care” to describe the intervention provided for the comparator arm of this trial.

The development of this intervention along with its acceptability among patients with major depressive disorder has been assessed by our team and thoroughly described elsewhere; this work is currently under review [25].

### The control (The Blues Breakers)

In addition to usual care, the control group will be offered a support group therapy format delivered at the same place, same visit frequency and same duration of program as the intervention group. This support group will be unstructured with no therapist involvement. Participants will be given the group meeting dates and time. Following this, a nurse with specialized training in research (education in data collection) who is not trained in behavioural activation will be present at the group time to collect study-related instruments and check the suicide risk question on the mood scales to ensure participant safety and clinical care. The participants will be asked to choose a topic of discussion, and one participant will lead the discussion. The choice of topic is entirely up to the participants.

We acknowledge that using an active control group has implications for the results of this work. The control group is receiving an enriched “usualcare” whereby patients enter an adjunct support group. Support groups are demonstrated as effective for improving quality of life [26], and thus, the implications of an active control arm will require consideration in the discussion and interpretation of findings for this pilot trial. This support group is intended to simulate the intervention group format to minimize risk of a biased estimate of BA effectiveness by reducing the potential placebo effect [27] which may result from frequent clinic visits and having additional attention beyond usual care.

#### Study question and hypothesis of the main study

In patients with depressive disorder attending a specialized hospital-based mood disorders clinic, does the addition of a behavioural activation program delivered in a group format decrease depressive symptoms and improve quality of life compared to treatment as usual and a support group after 18 weeks of treatment?

We hypothesize that group behavioural activation is an effective treatment for depressive disorder in patients with depression.

### Study objectives

#### Pilot study primary objectives

The main goal of the feasibility and pilot phase is to enhance the success of the full trial by testing the feasibility of conducting a randomized controlled trial to assess the effectiveness of a behavioural activation program for depression delivered through a group-based, face-to-face intervention in addition to care as usual to reduce depressive symptoms and improve quality of life. In addition, by using a qualitative study component, this study will also assess engagement in treatment among patients with depressive disorders and modify the protocol for the main trial based on the feedback received by the participants in the pilot phase. Engagement will be evaluated during the qualitative stage using open-ended questions aimed at determining patient satisfaction with the current treatment design. Engagement will also be evaluated during the BA program using assessment of attendance and involvement in group. In addition, the qualitative study aspect aims to collect data relating to participants’ experience in the program, as well as their perceptions of improvements in mood and functioning. This qualitative data will provide feedback to shape the main trial protocol.

The pilot primary objectives are therefore summarized after Thabane and colleagues [16]:

1. Assess the feasibility of the study process in terms of recruitment, retention, number of sessions completed, average group size and data completion
2. Assess resources needed including the use of interview rooms, group room, mobile computers to upload activity-tracking data, communication with participants’ clinicians and time investment in the program by the study clinicians.
3. The objective of the qualitative component of the pilot study is to ensure that the treatment program is patient-centred by modifying the protocol for the main trial as suggested by participants. More specifically, the qualitative study objectives are:
  - Solicit patient and clinician feedback on the behavioural activation program so that the program can be modified to meet the needs and preferences of the patient participants. Data collected towards this objective will help inform future programs and future research on this particular program of behavioural activation.
  - Throughout the study period, collect information from participants in the intervention and control arms of the study about their perceptions of their depression and quality of life in general. Collecting this information throughout the study from both sets of participants will allow us to compare and contrast the patients’ individual perspectives of the changes that they are experiencing. We will be able to compare this analysis with the quality of life measures to determine if there are additional domains of the illness experience affected by the behavioural activation program that are not covered by existing measures. These interviews throughout the study period also provide an opportunity to establish whether patients feel that their needs are being met, and whether they feel the program is responsive of such needs.

### Pilot study secondary objectives

1. Assess the change in mood using the Beck Depression Inventory between and within the intervention and control groups
2. Explore the change in quality of life scores between and within the intervention and control groups

### Trial Methods/design

This pilot study is a mixed-methods design including an open label pragmatic randomized trial and a qualitative study of participants’ experiences. The quantitative component of the study includes gathering data on demographics, recruitment rate, data completion, retention in pilot trial and changes in mood and quality of life measures. The qualitative component will be used to gather participants’ experiences, beliefs and opinions about two topics: (1) the experience of participating in the behavioural activation program or the support group (control condition) and (2) their evolving perceptions of their individual depressive illness and quality of life. Based on the analysis of qualitative data pertaining to the first topic, the behavioural activation program may be modified for the main trial. The qualitative study will involve multiple interviews and focus groups which will take place before, during and after the pilot intervention period. Participants will be drawn from both the intervention and control arms of the pragmatic randomized controlled trial. More details about the qualitative study can be found below.

The pilot pragmatic randomized controlled trial study design is a parallel 1:1 allocation comparing behavioural activation plus usual care to support group plus usual care. For this pilotstudy we will adopt the following principals simulating naturalistic real-life clinical setting to test the study question based on the pragmatic design [28, 29]:

1. No restrictive inclusion criteria will be used. Adults with major depressive disorder will be asked to participate in this study
2. Clinicians will deliver the BA program to participants randomized to receive the intervention
3. The intervention will be an add-on to usual care
4. The comparison group will receive usual care that may include medications, CBT and other therapies as required and decided by their clinical care
5. The primary outcome is patient-important (improvement in depressive symptoms and quality of life)
6. There will be no measures to improve adherence to the study intervention or the comparator.

### Trial design characteristics

**Fig. 1:**
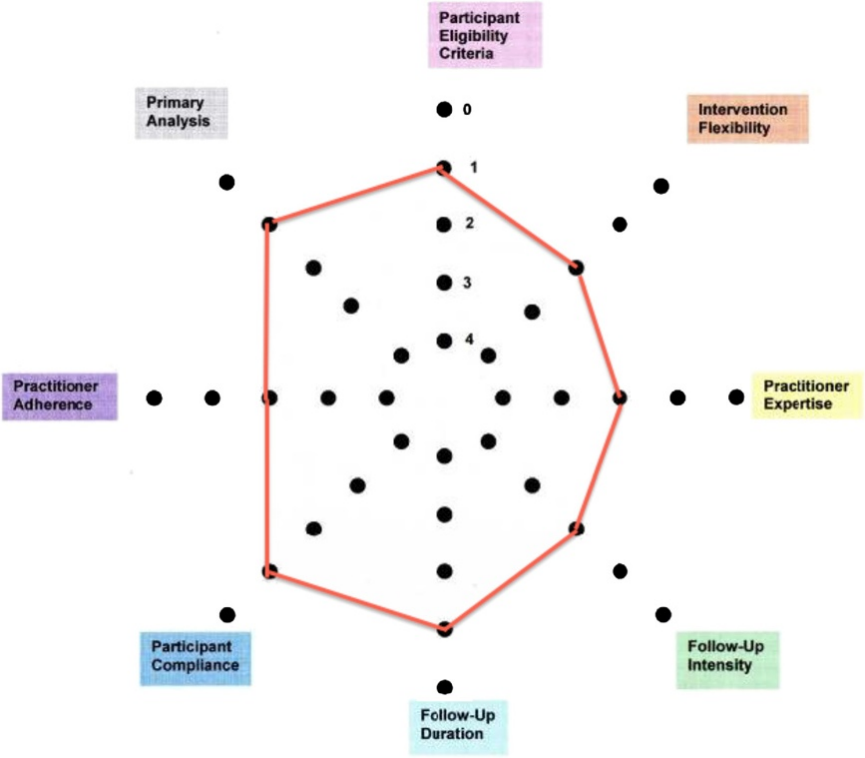
BRAVE pilot pragmatic trial design. The PrECIs tool shows the current pilot study design to be closer to a pragmatic than explanatory trial (http://www.cmaj.ca/content/180/10/E47.full). The red line represents the score for each domain “spoke” of the proposed BRAVE trial

We assessed the characteristics of the proposed trial using the pragmatic-explanatory continuum indicator summary (PRECIS) [30] on sample size, and outcome measures, eligibility criteria, flexibility of intervention administration, degree of practitioner experience needed for those applying the intervention and flexibility of administration of the comparator intervention from included trials. PRECIS is an accepted tool for assessing the design of trials on a continuum of effectiveness versus efficacy. Using the PRECIS criteria [30], Fig. 1 shows the current trial design based on the eight criteria “spokes” adapted from (http://www.support-collaboration.org/precis.pdf).

### Study setting

The study setting is an outpatient specialized mood disorders clinic at the Mood Disorders Program, St. Joseph’s Healthcare Hamilton. This is a single site study; however, the patients attending the mood disorders clinic are likely to represent the most severe end of the spectrum as this clinic acts as a tertiary care centre receiving referral from the Greater Hamilton and surrounding areas for consultation and management of patients with inadequate response to treatment.

### Clinicians providing BA (Out of the Blues group)

All clinicians (KM, KL and JW) involved in the intervention and administration of the study BA program work at the Mood Disorders Program and are trained in the use of BA for treatment of depression. All clinicians completed a workshop on BA in April 2013 and read three BA workbooks as training manuals (Michael Addis and Christopher Martell. Overcoming Depression One Step At A time, the new behavioural activation approach to getting your life back 2004; Jonathan Kanter, Andrew Busch and Laura Rusch. Behavioural Activation 2009; and Christopher Martell, Sona Dimidjian and Ruth Herman-Dunn. Behavioural Activation for Depression, a clinician guide 2010).

### Clinicians supervising control group (The Blues Breakers group)

Clinicians working at the Mood Disorders Program who provide usual care to patients attending the clinic will supervise the control group sessions. Clinicians running the control group have no training in behavioural activation.

All study participants (intervention and control groups) will continue to receive treatment as usual during their study participation. Information on the treatment received as part of usual care will be collected.

### Eligibility criteria

Adult patients (18 years or older) with major depressive disorders will be asked to participate in this study. Patients referred to the Mood Disorders Program and receiving treatment for depression as per usual clinical care including antidepressants medications, individual psychotherapy, cognitive behaviour therapy and other treatment modalities are eligible to participate in this study. All participants must be able to provide written informed consent and can attend the program sessions at the Mood Disorders Program. We will exclude patients who are unable to understand written and spoken English (the intervention is based on a group with English-speaking therapists and manuals) and who have a primary diagnosis other than depression. We acknowledge that placing a language restriction on the eligibility criteria may impact the generalizability of findings from this trial. However, while Hamilton may be pragmatically multilingual by nature, the delivery of services in this area (as well as the majority of Canada notwithstanding Quebec and New Brunswick) is delivered in English or with the use of a translator. In this study, we aimed to demonstrate changes in symptoms of depression and quality of life measures under optimal conditions; however, we may consider evaluating its effectiveness via translator at a later stage.

### The intervention

**Fig. 2:**
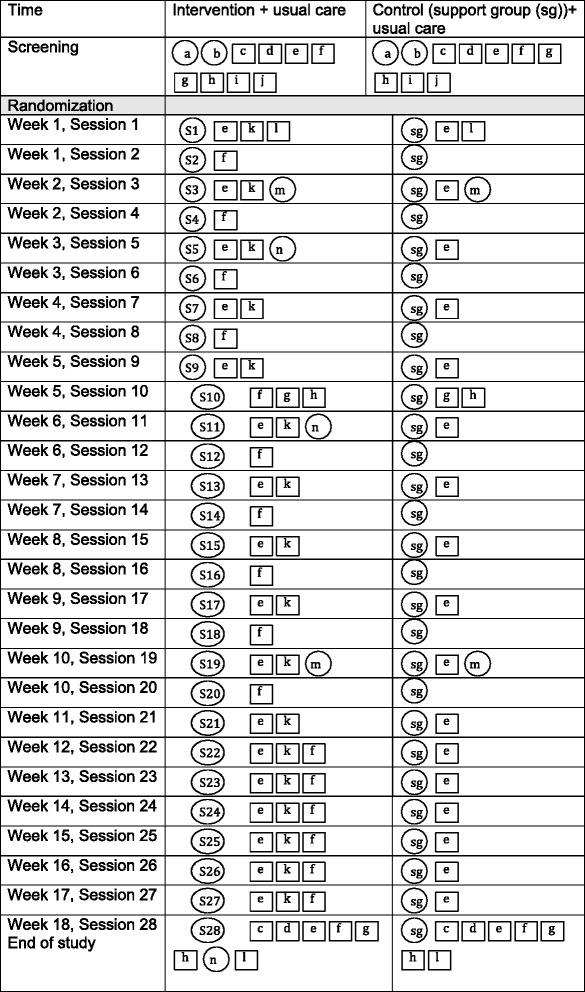
Pilot study components. a-eligibility and consent, b-baseline interview, diagnosis and demographics, c-physical measurements (height, weight, body fat percent), d-blood draw, e-Beck Depression Inventory (BDI II), f-Behavioural Activation for Depression Scale (BADS), g-Quality of life scales (SF-12, WSAS, and Q-LES-Q-SF), h-Leisure Motivation Scale (LMS), i-assumptions of risks, j-Physical Activity Readiness Questionnaire (PAR-Q), k- Activity tracking form, l- EQ-5D-5L a standardized instrument to measure health outcomes, m-qualitative individual interviews, n-focus group. S1-behavioural activation for depression, S2-values assessment, S3-goal setting, S4-Breaking it down, S5-avoidance and depression, S6- rumination, S7-ruminations II, S8-relationships, S9-involving others and social skills, S10-asseriveness and communication skills, S11-assetiveness and relaxation strategies, S12-leisure education, S13-problem solving, S14-team building and cooperative games, S15-nutrition and laughter yoga, S16- mindfullness and planning of group outing, S17-understanding sleep, S18-group outing, S19-getting back to work and volunteering, S20-S23 and S25-27 relapse prevention and troubleshooting, S24-booster group/adventure based day trip, S28-booster session and end of study. The qualitative components (l and m) are delivered in the pilot phase only. Adapted from Perera et al. [31]

Figure 2 describes the components of the intervention (adapted from Perera et al. [31]), number of sessions and time line of the pilot study. A specific intervention manual including detailed session-by-session administration of BA and complementary measures has been designed based on clinicians’ training, existing literature and BA manuals; the Out of the Blues intervention manual is available from authors upon request. In addition, the questionnaires and study instruments selected for the pilot trial are presented in Table 1. These instruments are as recommended by the BA manuals and previous literature on assessing treatment effectiveness in depression.

**Table 1:** Pilot study instruments

Instrument	Purpose	Administered	Time to complete (min)	When to complete	Intervention	Control
Baseline questionnaire	Baseline data	Clinician/research assistant	60	Baseline	✓	✓
PAR-Q	Assess readiness for physical activity	Self	10	Baseline	✓	
Assumption of risk form	Inform about risks of attending program	Self	5	Baseline	✓	
BDI-II	Monitor depressive symptoms	Self	5	Baseline	✓	✓
				Weekly	✓	✓
				End of study	✓	✓
SF12, WSAS, Q-LES-Q-SF	Quality of life	Self	10	Baseline	✓	✓
				Session 10	✓	✓
				End of study	✓	✓
BADS	Measure activation and avoidance behaviours	Self	5–10	Baseline	✓	✓
				Weekly	✓	
				End of study	✓	✓
LMS	Measure motivation for engaging in leisure activities	Self and clinician (observational)	15–30	Baseline	✓	✓
				Session 10	✓	
				End of study	✓	✓
EQ-5D-5 L	Health-related quality of life to generate health utility index for calculation of quality-adjusted life years for economic evaluation	Self	5	Baseline	✓	✓
				End of study	✓	✓
Activity-tracking form	To track activity	Clinician and patient	5	Weekly	✓	

### Outcomes

#### Pilot study outcomes

*Primary*:

1. Assess the feasibility of the study process in terms of recruitment, retention, number of sessions completed, average group size and data completion.
2. To assess resources needed including the use of interview rooms, group room, mobile computers to upload activity-tracking data, communication with participants’ clinicians and time investment in the program by the study clinicians.
3. The qualitative component of the pilot study is to assess the need to modify the protocol for the main trial based on the participants’ feedback thus aiming to provide a patient-centred treatment program.

*Secondary*:

1. Assess the change in mood using the Beck Depression Inventory between and within the intervention and control groups.
2. Explore the change in quality of life scores between and within the intervention and control groups.

### Timeline

The pilot study will be conducted over 18 weeks with a total of 30 visits including 28 sessions of behavioural activation in the intervention group and 28 sessions of support group in the control group. Both groups will also receive usual care. The study timeline is presented in more detail as in Fig. 2.

### Sample size

For the feasibility and pilot trial, we will recruit 10 participants in each arm. This number is based on the recommended group therapy size of 6–12 per group session. The pilot data will help to modify the sample size calculation for the main trial.

### Recruitment

Patients with a diagnosis of depressive disorders referred to the mood disorders clinic will be approached for participation in the study. Consecutive patients attending the clinic will be asked if they are interested in participating in the study. Following initial screening for eligibility, patients will be asked to provide written informed consent prior to any study-related procedures. Potential participants will be approached through clinicians with direct clinical contact with potential participants. A simple log will be kept to track the number of individuals approached, number declined, and number excluded and reasons for exclusion.

### Allocation and randomization

We will employ a parallel group design to test the feasibility of behavioural activation study in depression. Eligible and consenting patients will be randomly allocated to the intervention or control arms using a 1:1 allocation ratio. Allocations will be randomly assigned using a block randomization system of block sizes of 2, 4 and 6.

### Blinding and concealment of randomization

This pilot trial is an open label trial as blinding is not possible for participants (behavioural activation intervention) or the clinician administering the intervention. However, a research assistant not involved in the intervention or the control condition will allocate the participants based on the randomization system provided. The clinicians referring participants, the participants at the time of enrolment (signed consent and baseline visit completed) and the clinicians providing the BA intervention or the control groups will have no knowledge of the allocation prior to the start of the first intervention or control groups. Following the consent and baseline assessment, participants will be informed what group they will be attending. The clinicians will receive a list of the participants to attend their respective groups. Allocation concealment will be ensured by assigning study ID numbers to all participants at the time of enrolment and before randomization. The allocation then occurs as above using the assigned ID numbers with no other identifiers; these IDs will then be placed in an opaque envelope and randomly picked from the envelope and allocated to the intervention or control based on the block randomization system. Following the completion of the random allocation process, the ID numbers will then be identified by name, and the participants will be informed of their assigned groups. Further allocation concealment will occur at the data analysis level where data analyst will be blinded to the allocation. The clinical outcomes measures are self-administered, and therefore, the participants cannot be blinded to their allocation group.

### Data collection methods

Full description of the study instruments and timeline of administration are given in Table 1. A specifically designed case report form (CRF) will be used to collect the data using paper forms. A defining characteristic of pragmatic trials includes the use of simple easy-to-administer case report forms and psychometric tools. To ensure the feasibility of our baseline assessment instruments, we have elected to include as few psychometric tools as possible. For instance, we are only using a single depression scale and quality of life measure. The BDI-II includes 21 items measured on a scale of 0–3, with higher scores indicating a higher depressive symptom severity [32]. The BDI-II score is analyzed using four categories of depressive symptom severity [32]. We have chosen the BDI-II for two purposes. Firstly, the BDI-II will measure depression severity among participants [33]. The BDI-II identifies prognostically relevant depressive symptoms, such that it strongly predicts a variety of patient-important outcomes for populations suffering with depressive disorders [34]. Patient-important outcomes correspond to meaningful endpoints which identify closely with the patients values and preferences within this population, which may include physical or emotional symptoms, quality of life or social factors such as ability to obtain employments. Secondly, the BDI-II was chosen as a safety measure for the trial which is described later in the data-monitoring section of the trial protocol.

### Qualitative data collection

Using the methodology of Grounded Theory [35, 36], we will engage in an iterative data collection and analysis process to address our two qualitative research objectives.

Research objective (a) will be pursued by collecting focus group data from patient participants in the intervention and control arms and from clinicians involved in those patients’ care.

We have chosen to use focus groups for this objective to enable participants to build off of each other’s ideas and respond to the feedback shared by the other participants. These focus groups ask participants to comment on their shared experiences as a group, and therefore, the focus group is an appropriate method of data collection [37, 38]. Additionally, since the intervention being studied is a group-based program that aims to increase interaction and communication skills of patient participants, a focus group setting will be familiar to the patient groups. We will conduct homogeneous focus groups composed of either 4–7 patients or 4–7 clinicians at 3 time points: during weeks 3, 6, and 18 of the program. All program participants and their clinicians will be invited to participate, and anyone who is willing will be able to participate in a focus group. Focus groups will be conducted by a trained qualitative researcher who is not a clinician involved with patient care. Focus groups will be audio-recorded, and those recordings will be professionally transcribed for analysis [39]. A second researcher will take field notes during the focus group, noting body language, group dynamics and other aspects which are not recorded on the audiotape [37]. We will not be collecting this information (body language) for later use in a discourse analysis. Since we are administering a new intervention, it is important that we record all aspects of face-to-face contact, including during the qualitative sessions. The question guide for the focus groups will evolve throughout the process to reflect the emerging theory but will focus on the participant’s experience of the program and actively solicit constructive feedback to improve the intervention and ensure that it is as acceptable as possible to both patients and clinicians [35, 40]. For example, we might ask patients if the number of visits per week is manageable, if the timing of the program is acceptable, what barriers they experience to attending, what activities they find are particularly helpful or unhelpful etc. The focus group and interview guides are available from authors upon request. We might ask clinicians about their perceived need for this type of program for their patients, their willingness to refer patients to the program and whether they have observed a change in their patient since beginning the program.

Research objective (b) will be accomplished through individual interviews with patients (all patients will be invited to participation in these interviews) from both the control and intervention arms of the randomized study. Each patient will be interviewed twice, during weeks 2 and 10 of the program. Individual interviews were chosen to allow the participant the confidentiality to speak frankly about personal and sensitive issues. Participants will be asked about their perceptions of their depression and how it affects their quality of life, in whatever way they define quality of life. Participants will be asked whether they feel the program is tailored towards their needs and what changes should be made to address such needs. Answers to these questions will allow us to ensure that the program is patient-centred. Congruent with Grounded Theory, we will use purposive and theoretical sampling, and data collection will conclude when theoretical saturation is reached [35]. Theoretical saturation means that additional data collection does not yield new ideas or themes [35]. The individual interviews will be audio-recorded and professionally transcribed [39].

### Data management

Data from CRFs will be entered into research Electronic Data Capture (REDCap) (http://project-redcap.org/). Paper copies of the data will be stored securely as source documents, and electronic data will be hosted in the local institution server with passcode protection and electronic security measures in keeping with institutional policy and privacy regulations. Reports will be generated weekly to check data quality and missingness, whereby we will determine which variables are not being collected or captured properly.

### Statistical analyses

Data generated from the pilot study will help inform the main trial by testing the study procedures. Therefore, no formal statistical analyses will be performed for the pilot data. Description of the study participants and simple tabulation of the pilot data will be presented. Data exploration will be performed to compare the mean difference (and 95 % confidence intervals) of the BDI-II and quality of life scores between intervention and comparator groups at the end of the trial using *t* test and within groups for repeated measures using analysis of variance (ANOVA). All randomized participants will be included in the analyses. The statistical analyses of the pilot data will be exploratory only as the sample size will not allow for definitive analyses. We will use Stata version 12 software for analyses.

Summary of the study objectives and proposed statistical analyses are presented in Table 2. We will consider the following criteria to deem the study is feasible to be conducted:

1. At least 20 % of potential participants agree and enrol in the study (given the time commitment of 18 weeks and frequency of face-to-face visits we would expect fewer participants to enrol)
2. Completion rate of 80 %, that is to say, we will have final visit data on at least 80 % of the participants
3. We expect 80 % completion of weekly measurement scales

**Table 2:** Summary of the study objectives, outcomes and analysis plans

Aim	Objectives	Outcome	Hypothesis	Statistical analysis
Primary	Assess feasibility of recruitment, retention, group size and data completion	Recruitment and retention rates, data missingness	BA is feasible and acceptable	Descriptive statistics: mean and SD for continuous variables and proportions for dichotomous variables
	Assess resources needed	Group and interview rooms needed, number of clinicians to deliver groups, average length of group session (time needed per session)		Descriptive statistics: mean and SD for continuous variables and proportions for dichotomous variables
	Participants’ feedback and personal experiences	Qualitative study feedback		Qualitative study results based on grounded theory
Secondary	Clinical outcomes: BDI-II and quality of life	Rate of completion of the scales	The intervention group will show improvement in these scales	Between and within group comparisons of the BDI-II and quality of life scales

### Qualitative data analysis

The focus group sessions will be recorded using three devices in efforts to ensure that data are properly captured and to avoid any recording malfunction. Conversations will be transcribed in real-time by co-facilitators, and audio recordings will be used to verify these transcriptions. Facilitators will take extensive field notes, track participant responses, and ensured data are adequately collected from research participants.

We will use the analytical techniques of grounded theory, including line-by-line coding, thematic coding and constant comparative analysis [35, 41, 42]. These techniques require the analyst to look at small pieces of the data and then group and re-group the data into different categories. Additional data requires the entire dataset to be re-read and re-analyzed, to ensure the categories are still comprehensive and relevant. Data analysis will begin as soon as the first data are collected, and emerging findings will inform future data collection [35]. Three members of the research team individually reviewed the transcripts of the focus group to inductively identify themes before further analysis of the data. After further coding of the identified themes, they created an inclusive master list of themes. Dissenting opinions will be discussed and resolved. NVivo 9 software will be used to facilitate data management and analysis.

### Data monitoring

The main concern for this type of study is the presence of suicidal risk in the intervention and control groups. Suicide risk is increased in depressive disorders, and a specific question in the depression symptoms score using BDI-II is dedicated to suicidal ideas. Therefore, monitoring this specific question is important during the study. For this reason, the BDI-II questionnaires will be completed at the clinic and the answers checked by the clinician while the participants are in the program. If the suicide question is checked positive, the patient will be asked to stay in the clinic, and the patient psychiatric care provider will be informed. This is a common practice for clinical and research processes taken place at the mood disorders clinic. The use of BDI-II is also a common practice, and all clinicians are familiar with scoring and checking the suicide question.

This study does not require a data and safety monitoring board due to the reasons described above in a social/behavioural research type of study.

### Harms

This study is a minimal risk non-pharmaceutical study; however, there may be risks involved including anxiety or fatigue when completing the study questionnaires, interviews and attending groups. In addition, participants allocated to the control group may feel disappointed that they did not receive the new intervention. The study investigators interviewing participants and administering intervention/control conditions are experienced clinicians delivering psychiatric care at the clinic; in addition, all participants will also continue to have usual care. The study clinicians will be in close contact with participants’ clinical teams. If participants are seen in distress, fatigue or anxious situations, the clinician will attend to the participant needs and remove them from the group/study if necessary.

### Auditing

The study team will meet weekly to discuss the study progress and review the weekly report of study recruitment, data quality and monitoring of attendance and any issues raised by the participants or the clinicians.

### Research ethics approval

Approval from the Hamilton Integrated Research Ethics Board (HIREB) has been obtained.

### Protocol amendments

Any changes to the study protocol (expected during and after the completion of the pilot phase) will be reported to the HIREB prior to implementation. In addition, any protocol deviations/violations will be reported promptly, and a note to file will be kept in the study file.

### Consent

Informed consent will be obtained during a face-to-face interview with potential participants by a member of the research team in a private office within the hospital premises. Details of the study procedures and time commitment will be explained verbally and reviewed with the potential participants for any questions. Any questions arising will be answered accordingly. The informed consent form will be reviewed page by page with each potential participant. Participants will be informed that they can request additional time to consider participation and take a copy of the consent form to review at their leisure before making a decision on enrolling in the study.

### Confidentiality

All study personnel will be trained and monitored regularly in the requirement of participants’ confidentiality according to hospital and research ethics board regulations and following good clinical practice guidelines. All research-related procedures including data collection and storage will be carried out in secure clinical and research-designated areas within the hospital property. No information about any participants will be shared outside the research team without prior consent unless there are concerns regarding participant health and safety, and in this instance, these concerns will be communicated to the participant and their clinician.

### Access to data

Study investigators will have access to the pilot trial data.

### Post trial care

All study participants have access to care at the Mood Disorders Program during the pilot trial and will continue to receive usual care at the conclusion of the pilot trial. The pilot trial procedures do no replace any existing services that the participants are otherwise receiving.

### Dissemination

The Mood Disorders Program website will be used to introduce the behavioural activation program. In addition, participants will be informed on the main trial progress through a newsletter that will be posted on website and mailed to participants yearly.

The trial results will be shared with healthcare professionals through grand rounds presentations, scientific meetings and peer-reviewed publications.

For the public at large, the trial results will be presented at the annual Mood Disorders Program Tackling Depression event hosted once per year to disseminate mood disorder-related topics to health care providers within the community and the general public.

The trial reporting will follow the CONSORT statement extension for pragmatic trials [29]. The study protocol follows the SPIRIT guidelines [43].

## Discussion

This study protocol will help to implement the pilot and ultimately the main trial in a transparent way to assist in improving the reporting and conduct of non-pharmaceutical clinical trials. The pilot study will show if the program of behavioural activation is feasible to be delivered at the planned frequency and duration of intervention. The pilot study will also provide insight into the feasibility of an 18-week follow-up for patients with major depressive disorder. Provided we demonstrate that an 18-week follow-up is feasible, we may consider the additional time needed to establish effectiveness among this patient population.

In addition, the feedback from the participants will be important to design patient-focused interventions that are likely to be used by participants in the future. We anticipated challenges during the pilot study to include the time commitment to the intervention and the face-to-face visits to the hospital. Concerns regarding the time, cost (for example parking/transportation) and attendance of the control group as they will be aware that the group they are allocated to is the control group. If such challenges were to arise, we will attempt to overcome these challenges, we will offer public transportation and parking costs for all participants and we will offer behavioural activation to the control group once the pilot trial is completed.

## Abbreviations

BA: behavioural activation
BDI-II: Beck depression inventory II
CBT: cognitive behavioural therapy
CRF: case report form
EQ-5D-5L: EuroQol 5-Dimension

## References

[1] Wild B, Herzog W, Schellberg D, Lechner S, Niehoff D, Brenner H, Rothenbacher D, Stegmaier C, Raum E (2012). Association between the prevalence of depression and age in a large representative German sample of people aged 53 to 80 years. Int J Geriatr Psychiatry.

[2] Martin-Merino E, Ruigomez A, Johansson S, Wallander MA, Garcia-Rodriguez LA (2010). Study of a cohort of patients newly diagnosed with depression in general practice: prevalence, incidence, comorbidity, and treatment patterns. Prim Care Companion J Clin Psychiatry.

[3] Wedegaertner F, Arnhold-Kerri S, Sittaro NA, Bleich S, Geyer S, Lee WE (2013). Depression- and anxiety-related sick leave and the risk of permanent disability and mortality in the working population in Germany: a cohort study. BMC Public Health.

[4] Pigott HE, Leventhal AM, Alter GS, Boren JJ (2010). Efficacy and effectiveness of antidepressants: current status of research. Psychother Psychosom.

[5] Sicras-Mainar A, Maurino J, Cordero L, Blanca-Tamayo M, Navarro-Artieda R (2012). Assessment of pharmacological strategies for management of major depressive disorder and their costs after an inadequate response to first-line antidepressant treatment in primary care. Ann Gen Psychiatry.

[6] Colman I, Naicker K, Zeng Y, Ataullahjan A, Senthilselvan A, Patten SB (2011). Predictors of long-term prognosis of depression. Can Med Assoc J.

[7] Huhn M, Tardy M, Spineli L, Kissling W, Förstl H, Pitschel-Walz G (2014). Efficacy of pharmacotherapy and psychotherapy for adult psychiatric disorders: a systematic overview of meta-analyses. JAMA Psychiatry.

[8] Sudak DM (2012). Cognitive behavioral therapy for depression. Psychiatr Clin North Am.

[9] Martell CR, Dimidjian S, Herman‐Dunn R (2010). Behavioral activation for depression: a clinician’s guide.

[10] Lynch D, Laws KR, McKenna PJ (2010). Cognitive behavioural therapy for major psychiatric disorder: does it really work? A meta-analytical review of well-controlled trials. Psychol Med.

[11] Høifødt RS, Strøm C, Kolstrup N, Eisemann M, Waterloo K (2011). Effectiveness of cognitive behavioural therapy in primary health care: a review. Fam Pract.

[12] Mazzucchelli T, Kane R, Rees C (2009). Behavioral activation treatments for depression in adults: a meta-analysis and review. Clin Psychol Sci Pract.

[13] Blenkiron P (1999). Who is suitable for cognitive behavioural therapy?. J R Soc Med.

[14] Morrison N (2001). Group cognitive therapy: treatment of choice or sub-optimal option?. Behav Cogn Psychother.

[15] Trivedi MH, Rush AJ, Wisniewski SR, Warden D, McKinney W, Downing M, Berman SR, Farabaugh A, Luther JF, Nierenberg AA (2006). Factors associated with health-related quality of life among outpatients with major depressive disorder: a STAR*D report. J Clin Psychiatry.

[16] Thabane L, Ma J, Chu R, Cheng J, Ismaila A, Rios LP, Robson R, Thabane M, Giangregorio L, Goldsmith CH (2010). A tutorial on pilot studies: the what, why and how. BMC Med Res Methodol.

[17] Veale D (2008). Behavioural activation for depression. Adv Psychiatr Treat.

[18] Kanter JW, Manos RC, Bowe WM, Baruch DE, Busch AM, Rusch LC (2010). What is behavioral activation? A review of the empirical literature. Clin Psychol Rev.

[19] Jacobson NS, Dobson KS, Truax PA, Addis ME, Koerner K, Gollan JK, Gortner E, Prince SE (1996). A component analysis of cognitive-behavioral treatment for depression. J Consult Clin Psychol.

[20] Kanter JW, Bush AM, Rusch LC (2009). Behavioural activation: the CBT distinctive feature series.

[21] Cuijpers P, van Straten A, Warmerdam L (2007). Behavioral activation treatments of depression: a meta-analysis. Clin Psychol Rev.

[22] Nierenberg AA, Ostacher MJ, Huffman JC, Ametrano RM, Fava M, Perlis RH (2008). A brief review of antidepressant efficacy, effectiveness, indications, and usage for major depressive disorder. J Occup Environ Med.

[23] Mitchell J, Greenberg J, Finch K, Kovach J, Kipp L, Shainline M, Jordan N, Anderson C (1997). Effectiveness and economic impact of antidepressant medications: a review. Am J Manag Care.

[24] Strauss DC, Thomas JM (2009). What does the medical profession mean by “standard of care?”. J Clin Oncol.

[25] Samaan Z, Dennis BB, Kalbfleisch L, Bami L, Zielinski L, Bawor M, et al. Behavioral activation for reducing depressive symptoms and improving quality of life: a feasibility study part one: a focus group study examining clinician and patient opinions on components of “Behavioural Activation Plus” Program. Under Review. 2015.

[26] Pfeiffer PN, Heisler M, Piette JD, Rogers MA, Valenstein M (2011). Efficacy of peer support interventions for depression: a meta-analysis. Gen Hosp Psychiatry.

[27] Wampold BE, Minami T, Tierney SC, Baskin TW, Bhati KS (2005). The placebo is powerful: estimating placebo effects in medicine and psychotherapy from randomized clinical trials. J Clin Psychol.

[28] Patsopoulos NA (2011). A pragmatic view on pragmatic trials. Dialogues Clin Neurosci.

[29] Zwarenstein M, Treweek S, Gagnier JJ, Altman DG, Tunis S, Haynes B, Oxman AD, Moher D (2008). Improving the reporting of pragmatic trials: an extension of the CONSORT statement. BMJ.

[30] Thorpe KE, Zwarenstein M, Oxman AD, Treweek S, Furberg CD, Altman DG, Tunis S, Bergel E, Harvey I, Magid DJ (2009). A pragmatic-explanatory continuum indicator summary (PRECIS): a tool to help trial designers. CMAJ.

[31] Perera R, Heneghan C, Yudkin P (2007). Graphical method for depicting randomised trials of complex interventions. BMJ.

[32] Wang YP, Gorenstein C (2013). Psychometric properties of the Beck Depression Inventory-II: a comprehensive review. Rev Bras Psiquiatr.

[33] Krefetz DG, Steer RA, Gulab NA, Beck AT (2002). Convergent validity of the Beck depression inventory-II with the reynolds adolescent depression scale in psychiatric inpatients. J Pers Assess.

[34] Edwards BC, Lambert MJ, Moran PW, McCully T, Smith KC, Ellingson AG (1984). A meta-analytic comparison of the Beck Depression Inventory and the Hamilton Rating Scale for Depression as measures of treatment outcome. Br J Clin Psychol.

[35] Charmaz K. Constructing grounded theory: a practical guide through qualitative analysis. Pine Forge Press; 2006. https://books.google.com.ph/books?id=w2sDdv-S7PgC&printsec=frontcover&source=gbs_ge_summary_r&cad=0#v=onepage&q&f=false

[36] Charmaz K, Holstein JA, Gubrium JF (2008). Constructionism and the grounded theory method. Handbook of constructionist research. edn.

[37] Kitzinger J. Qualitative Research: Introducing focus groups BMJ 1995; 311:299. 10.1136/bmj.311.7000.299 PMC2550365 7633241

[38] Sim J (1998). Collecting and analysing qualitative data: issues raised by the focus group. J Adv Nurs.

[39] Poland B (1995). Transcription quality as an aspect of rigor in qualitative research. Qual Inq.

[40] Charmaz K, Denzin NK, Lincoln YS (2000). Grounded theory: objectivist and constructivist methods. Handbook of qualitative research. Volume 2, edn.

[41] Boeije H (2002). A purposeful approach to the constant comparative method in the analysis of qualitative interviews. Qual Quant.

[42] Corbin J, Strauss A. Basics of qualitative research: techniques and procedures for developing grounded theory. Sage; 2008.

[43] Chan AW, Tetzlaff JM, Gotzsche PC, Altman DG, Mann H, Berlin JA, Dickersin K, Hrobjartsson A, Schulz KF, Parulekar WR (2013). SPIRIT 2013 explanation and elaboration: guidance for protocols of clinical trials. BMJ.

